# Effect of a Multifaceted, Church-Based Wellness Program on Metabolic Syndrome in 41 Overweight or Obese Congregants

**Published:** 2010-06-15

**Authors:** Priscilla Ivester, Susan Sergeant, Suzanne C. Danhauer, L. Douglas Case, Alec Lamb, Briana G. Chilton, Bonnie Delar, Monica L. Hollingshead, Floyd H. Chilton, Kelly L. Weaver

**Affiliations:** Center for Botanical Lipids; Wake Forest University Health Sciences, Winston-Salem, North Carolina; Wake Forest University Health Sciences, Winston-Salem, North Carolina; Wake Forest University Health Sciences, Winston-Salem, North Carolina; Wake Forest University Health Sciences, Winston-Salem, North Carolina; Wake Forest University Health Sciences, Winston-Salem, North Carolina; Wake Forest University Health Sciences, Winston-Salem, North Carolina; Wake Forest University Health Sciences, Winston-Salem, North Carolina; Wake Forest University Health Sciences, Winston-Salem, North Carolina; University of Miami School of Medicine, Miami, Florida. At the time of this research, Dr Weaver was affiliated with Wake Forest University Health Sciences, Winston-Salem, North Carolina

## Abstract

**Introduction:**

A rise in obesity, poor-quality diets, and low physical activity has led to a dramatic increase in the number of Americans with metabolic syndrome and diabetes. Our objective was to determine the effect of a short-term, multifaceted wellness program carried out in a church setting on weight, metabolic syndrome, and self-reported wellness.

**Methods:**

Forty-one overweight or obese adults in a church congregation provided fasting blood samples and answered a wellness questionnaire before and after completing an 8-week diet and exercise program. We also measured weight, body fat, body mass index, and waist and hip circumference.

**Results:**

The intervention decreased weight, body fat, and central adiposity; improved indexes of metabolic syndrome; and increased self-reported wellness.

**Conclusion:**

A multifaceted wellness intervention that emphasizes diet and exercise can rapidly influence weight, insulin resistance, metabolic syndrome, and self-reported wellness.

## Introduction

During the past 4 decades, the prevalence of obesity in the United States has increased dramatically. If these trends continue, 83% of adults will be overweight or obese by 2030 (1). Obesity is associated with various conditions (dyslipidemia, elevated blood pressure, and insulin resistance or glucose intolerance) that collectively are known as metabolic syndrome, which affects 50 to 75 million Americans (1,2).

Nutrition and physical activity are key lifestyle factors that affect the incidence of obesity, metabolic syndrome, and other diseases. Even modest weight loss improves dyslipidemia, insulin resistance, and estimated 10-year cardiovascular disease risk (3-7). Millions of Americans enroll in weight loss programs each year, but few such programs have been clinically tested. When they are tested, results generally show poor efficacy and adherence (8). Weight Watchers is the only weight loss program that has been tested extensively; it results in a mean loss of approximately 5% of initial weight in 3 to 6 months, but 70% of participants stop attending within 12 weeks (8).

Because wellness programs can reduce the cost of health care and generate a substantial return on investment (savings of $1.49 to $4.91 in health care costs per dollar spent on wellness) (9), programs are needed that focus on weight loss, metabolic syndrome, chronic disease prevention, and emotional well-being. Our goal was to determine whether a multifaceted diet and exercise program could result in weight loss and decrease insulin resistance, reduce risk for metabolic syndrome, and increase overall wellness in overweight and obese adults. The program was conducted in a church setting to foster motivation, accountability, and social support. We examined physical, biochemical, and psychological endpoints to assess the effect of the program on obesity and several comorbidities.

## Methods

### Participants

We recruited participants from the congregation of the Hillsdale United Methodist Church (Advance, North Carolina). Pregnant women were excluded because the program involved caloric restriction and moderate to vigorous exercise. Pregnancy was the only exclusion criterion; all other congregants who received permission from their primary care physician and provided written informed consent were allowed to participate. This study was conducted according to approved human studies guidelines and was approved by the institutional review board of Wake Forest University Health Sciences.

A total of 59 participants began the program, 13 of whom were healthy weight (body mass index [BMI] <25 kg/m^2^) and 46 of whom were overweight or obese (BMI ≥25 kg/m^2^). Healthy-weight participants did not restrict calories and were excluded from analysis; of the overweight or obese participants, 5 did not participate in the entire intervention and were also excluded from analysis.

### Intervention

At the beginning of the intervention, in April 2008, participants were given a lay language handout (approximately 50 pages) that described the dietary and exercise recommendations of the program. Participants also attended 2 weekly educational programs (approximately 5 hours total) in which the scientific rationale behind the recommendations was explained. The dietary recommendations had 5 components:

1. Calorie reduction. Overweight and obese participants were told to reduce calorie intake by 20% from their individual baseline as determined by a predictive equation for resting energy expenditure (10) for the first 4 weeks and an additional 10% thereafter. To assist in calorie reduction, a soup fast was recommended once weekly.
2. Increased fiber intake. A daily fiber intake of 16 g per 1,000 kcal consumed and a 70:30 insoluble-to-soluble fiber ratio was recommended.
3. Increased polyphenol intake. Fruits and vegetables high in *trans*-resveratrol, anthocyanins, and ellagic acids and green teas high in catechins were recommended. In the final 4 weeks, participants were allowed red wine (4-6 glasses/wk) and dark chocolate (43-99 g/d).
4. Increased intake of long-chain omega-3 fatty acids. Fatty fish and fish oil supplements were recommended (suggested dose, 1,250-1,500 mg/d eicosapentaenoic acid plus docosahexaenoic acid).
5. Increased intake of short-chain omega-6 fatty acids. Borage oil supplements were recommended (γ-linolenic acid, 400-500 mg/d). These doses and ratios of omega-3 and omega-6 fatty acids were previously determined in our laboratory (11).

Participants were provided with heart rate monitors and were encouraged to exercise at least 30 minutes per day at an appropriate level (50%-75% of maximum heart rate for age during weeks 1-3 and 70%-85% of maximum heart rate for age thereafter). Participants were told to dedicate 3 days per week to aerobic exercise and 2 days for circuit training, which consisted of aerobic warm-up and cool-down combined with weight training designed to work 8 to 10 muscle groups. The intensity of the recommended exercise increased from mostly light at the beginning of the intervention to mostly vigorous by the end. Instructions for specific aerobic workouts and circuit training were described in the program handout. Participants received instruction in performing each exercise at a level appropriate to his or her fitness level during the first weekly exercise session at the church. They were encouraged to purchase resistance bands and hand weights for use in their exercise routines. Exercise was undertaken independently and once weekly at the church.

Participants met weekly for body measurements, group exercise sessions at the church, dietary counseling, and group support. This church group took a faith-based approach to mutual support, encouragement, prayer, and striving to make their bodies healthy. Participants kept logs of their food intake and exercise to facilitate lifestyle changes and accountability.

### Data collection and analysis

At the beginning of the intervention, we collected baseline urine, fasting blood samples, anthropomorphic measurements (weight, percentage body fat, BMI, and waist and hip circumference), blood pressure, and resting heart rate. Thereafter, body fat and BMI were measured at weeks 1, 4, and 8. At the end of the 8-week intervention, all fasting samples and measurements were obtained again. Follow-up anthropomorphic measurements were obtained 5 and 10 weeks after the intervention. In addition to anthropomorphic measurements, 6 surrogate markers of insulin resistance and diabetes — fasting insulin, fasting glucose, homeostatic model assessment of insulin resistance (HOMA) (12), hemoglobin A1c (13), stearoyl-coenzyme A desaturase 1 activity, and circulating palmitoleic acid (14) — were measured. Participants were also asked to complete a medical history.

Throughout the study, the same person performed waist and hip measurements. Weight, body fat, and BMI were measured by using a Tanita BF-350 body fat scale (Tanita Corporation, Arlington Heights, Illinois). The North Carolina Baptist Hospital Clinical Laboratories (Winston-Salem, North Carolina) measured complete blood counts and differentials. Serum was separated, aliquoted, and frozen until used to measure levels of lipid, glucose, and other metabolites according to standard clinical laboratory methods or previously published methods (15).

At baseline and week 8, participants completed a wellness questionnaire, a modified self-rated health and wellness survey (16) designed to assess mental and physical health attitudes and health behaviors. This survey emphasized perception of wellness rather than disease symptoms by evaluating physical distress (3 items), emotional distress (8 items), emotional well-being (3 items), and perceived stress (7 items). The survey included items that assessed interest in maintaining a healthy lifestyle, sleep difficulties, body image (perception of physical appearance), and feelings about life as a whole.

We used paired *t* tests to assess changes in continuous variables. We used analysis of variance to determine whether the changes differed by sex. We used an exact McNemar test to assess changes in dichotomous variables (for example, prevalence of metabolic syndrome). All analyses were conducted by using SAS version 9.1 (SAS Institute Inc, Cary, North Carolina). We considered *P* values less than .05 to be significant.

## Results

Of 46 overweight or obese participants who began the program, 41 (27 women and 14 men) completed it. Participants' mean age was 53 years (range, 35-74 years), and all participants were white with the exception of 1 female African American participant. At baseline, all participants exceeded the healthy cutoff value for percentage of body fat (≤31% for women and ≤21% for men); 17 women and 13 men also exceeded the healthy cutoff value for waist-to-hip ratio (≤0.8 for women and ≤0.9 for men). Eighteen participants completed a medical history, and of these, 12 reported immunologic conditions (such as allergy, asthma, arthritis, or atopic dermatitis), 8 reported cardiovascular disease (such as high blood pressure or history of heart attack), 5 reported high cholesterol, and 2 reported diabetes.

During the 8-week intervention, men lost an average of 5.9 kg (13.1 lb) and women lost an average of 4.8 kg (10.5 lb), which is approximately a 6% decrease in body weight for both men and women (Figure). After the intervention, the participants' weights were stable for 5 weeks; at 10 weeks, women's weight remained stable but men began to regain weight.

**Figure.**
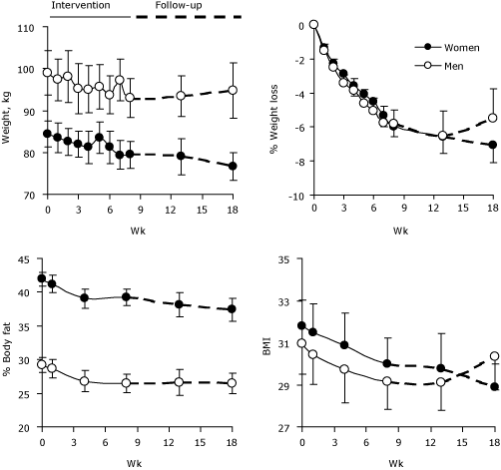
Weight, percentage of weight loss, percentage of body fat, and body mass index (BMI) in 41 overweight or obese congregants (27 women and 14 men) during an 8-week diet and exercise intervention and 10-week follow-up, Hillsdale United Methodist Church Wellness Study, Advance, North Carolina, April-August 2008. Data points are means and error bars represent standard errors.

**Table:** 

**Wk**	**Weight, kg**			

	**Women**		**Men**	

	**Mean**	**SE**	**Mean**	**SE**
0	84.3	3.1	98.9	5.2
1	83.5	3.4	97.4	5.0
2	82.6	3.2	98.0	6.2
3	82.0	3.1	95.1	6.0
4	81.2	3.8	95.1	5.5
5	83.5	3.7	95.6	5.3
6	81.2	3.8	93.7	4.5
7	79.4	3.5	97.2	5.0
8	79.5	3.2	93.0	4.6
13	79.0	4.2	93.4	4.9
18	76.7	3.3	94.8	6.7

**Wk**	**% Weight Loss**			

	**Women**		**Men**	

	**Mean**	**SE**	**Mean**	**SE**
0	0.0	0.0	0.0	0.0
1	−1.4	0.3	−1.5	0.3
2	−2.3	0.3	−2.5	0.5
3	−2.9	0.4	−3.4	0.5
4	−3.6	0.6	−3.9	0.7
5	−4.1	0.5	−4.6	0.8
6	−4.5	0.6	−5.0	0.8
7	−5.3	0.7	−5.8	1.0
8	−6.0	0.6	−5.8	0.8
13	−6.6	1.0	−6.5	1.5
18	−7.1	1.0	−5.5	1.7

**Wk**	**% Body Fat**			

	**Women**		**Men**	

	**Mean**	**SE**	**Mean**	**SE**
0	41.9	1.0	29.2	1.1
1	41.2	1.2	28.7	1.3
4	39.1	1.4	26.8	1.6
8	39.2	1.3	26.5	1.4
13	38.2	1.8	26.6	1.9
18	37.4	1.7	26.4	1.5

**Wk**	**BMI**			

	**Women**		**Men**	

	**Mean**	**SE**	**Mean**	**SE**
0	31.8	1.2	31.0	1.4
1	31.5	1.4	30.4	1.4
4	30.9	1.5	29.7	1.6
8	30.0	1.3	29.1	1.3
13	29.8	1.7	29.1	1.3
18	28.9	1.1	30.4	1.6

Similarly, body fat and BMI decreased in both men and women during the 8 weeks of the program. In women, these trends continued during the 10-week follow-up. In men, body fat remained stable during the follow-up period, but BMI began to increase after 5 weeks (Figure). Waist circumference, hip circumference, and waist-to-hip ratio also significantly decreased in both women and men during the intervention. By the end of the intervention, 6 overweight participants (4 women, 2 men) had moved into the healthy-weight BMI range, and 1 obese participant had moved into the overweight BMI range.

Twenty-seven participants (17 women, 10 men) turned in complete wellness questionnaires at both baseline and 8 weeks. Participants reported significant improvements in most domains (Table 1).

After the intervention, waist circumference, a measure of central adiposity, was significantly decreased in women and men, and systolic blood pressure was significantly decreased in men (Table 2). Most other risk factors for metabolic syndrome improved, but the differences did not reach significance. The average number of metabolic syndrome risk factors, however, did significantly decrease for women (from 2.0 to 1.6, *P* = .02) and men (from 2.5 to 1.5, *P* < .001). At baseline, 18 of 41 participants were classified as having metabolic syndrome (10 women and 8 men). After the intervention, that number had decreased to 10 (6 women and 4 men) (*P* < .001).

Although fasting glucose levels did not change (Table 2), all other markers of insulin resistance decreased in both women and men by the end of the intervention (Table 3).

## Discussion

This multifaceted intervention significantly improved the conditions that define metabolic syndrome and reduced the number of participants who had metabolic syndrome. We also found improvements in several measures of insulin resistance. These observations suggest that a multifaceted approach to wellness that incorporates nutrition education and exercise in a supportive, faith-based environment can promote healthy lifestyle changes in overweight and obese adults.

Our findings contrast with results of several commercial weight loss programs, which have high attrition rates, rapid weight regain, and poor adherence (8). More than 90% of our participants finished the 8-week intervention, and on average, women in our study continued to lose weight 10 weeks after the end of the program. Feedback indicated that reasons for adherence included the emphasis on behavioral modifications, realistic goals, information on how to meet goals, the hunger-curbing effects of fiber and water, regular group support and exercise, accountability to the group and its leaders, emphasis on disease management and prevention, use of diet and exercise logs, and the church setting where the group had common belief systems and tools.

Significant changes in indexes of metabolic syndrome and insulin resistance were achieved in a short time and in participants who lost only 6% to 8% of their original body weight and 9% to 13% of their original fat mass. This finding suggests that decreased fat mass is not the only factor responsible for the decreased insulin resistance. Whether the changes in metabolic syndrome were associated with those in insulin resistance is not clear. A recent study showed that metabolic syndrome is a significant predictor of diabetes, increasing the risk by a factor of 3.5 to 5.2 (17); this association is significantly stronger than the association of metabolic syndrome with cardiovascular events (18).

Our intervention incorporated several dietary and exercise strategies. Consequently, determining the precise mechanisms responsible for the improvements is difficult. Several studies have found that exercise independently decreases glucose levels and risk of type 2 diabetes (19,20), and calorie restriction independently lowers insulin and triglyceride levels (19,21). In the CALERIE study, fasting insulin levels were significantly reduced from baseline at months 3 and 6 in both the calorie restriction group and the calorie restriction plus exercise group (19). The Diabetes Prevention Program showed that lifestyle changes, including exercise, for at least 2 years reduced the incidence of diabetes in high-risk populations (5).

The specific dietary recommendations in this study likely contributed to the observed improvements. A high-fiber diet, as recommended in this study, improves glycemic control (22,23), and adding long-chain omega-3 fatty acids to diets reduces risk of the components of metabolic syndrome (especially hyperlipidemia) and cardiovascular disease (24). Additionally, several studies indicate that adding polyphenols to diets decreases insulin resistance and reduces risk of metabolic syndrome (25-27). In our study, glucose regulation rapidly improved, most likely because the components of the intervention acted in an additive or synergistic manner.

This study has several limitations. First, the program was conducted at only 1 church, so the results cannot be generalized and the program may not be feasible in other settings. The efficacy of the program should be evaluated in groups that differ in age and ethnic and racial composition. Second, because of the multifaceted nature of the intervention, we cannot elucidate specific aspects that affected insulin resistance or metabolic syndrome. Third, physical and emotional wellness were assessed by self-report; however, self-perception of wellness is a complex sum of individual health values and social and environmental influences (28), all of which may vary among different ethnic and racial populations. Fourth, we did not include a control group, so we cannot say what changes would have occurred in people who did not undergo the intervention. Finally, our sample size was small and limits our power to detect smaller changes in some of the outcome measures.

In conclusion, we found that a multifaceted, church-based wellness program that emphasizes education, a healthy diet, weight loss, physical activity, weekly coaching, and accountability can rapidly (within 8 weeks) decrease insulin resistance and risk of metabolic syndrome.

## Figures and Tables

**Table 1:** Effect of an 8-Week Diet and Exercise Intervention on Self-Reported Wellness and Quality of Life, Hillsdale United Methodist Church Wellness Study, Advance, North Carolina, April-August 2008

Domain	Women (n = 17), Mean ± SD			Men (n = 10), Mean ± SD		

	Baseline	Week 8	*P* Value^a^	Baseline	Week 8	*P* Value^a^
Physical distress^b^	3.2 ± 0.8	2.4 ± 0.6	.005	2.7 ± 0.8	2.2 ± 0.5	.04
Emotional distress^b^	2.6 ± 0.6	2.1 ± 0.3	.001	2.1 ± 0.4	1.8 ± 0.4	.009
Perceived stress^b^	2.4 ± 0.8	2.0 ± 0.5	.01	2.2 ± 0.6	1.9 ± 0.4	.04
Sleep^b^	2.4 ± 0.9	2.0 ± 0.8	.21	2.4 ± 0.8	2.1 ± 0.7	.34
Body image^c^	3.9 ± 1.5	4.8 ± 1.2	.02	3.5 ± 1.4	5.1 ± 1.0	.001
Life as a whole^c^	5.3 ± 1.2	5.5 ± 1.1	.30	5.4 ± 0.8	6.1 ± 0.7	.001
Emotional well-being^d^	3.2 ± 0.7	3.6 ± 0.7	.002	3.4 ± 0.5	3.9 ± 0.7	.06
Healthy lifestyle^d^	4.4 ± 0.6	4.8 ± 0.4	.004	3.5 ± 1.0	4.5 ± 0.5	.008

Abbreviation: SD, standard deviation.

a Pairwise analysis of baseline vs week 8 differences for each group (women, men) by using paired *t* test (2-tailed).

b Range, 1-5; lower scores indicate better well-being.

c Range, 1-7; higher scores indicate better well-being

d Range, 1-5; higher scores indicate better well-being.

**Table 2:** Effect of an 8-Week Diet and Exercise Intervention on Risk Factors for Metabolic Syndrome, Hillsdale United Methodist Church Wellness Study, Advance, North Carolina, April-August 2008

Risk Factor^a^	Women (n= 27)				Men (n= 14)			

	Baseline		Week 8^b^		Baseline		Week 8^b^	

	Mean ± SD	No. With Risk	Mean ± SD	No. With Risk	Mean ± SD	No. With Risk	Mean ± SD	No. With Risk
Waist circumference, cm	93.7 ± 15.5	14	82.8 ± 14.5	7	109.2 ± 19.2	10	97.8 ± 11.6	4
Triglyceride level, mg/dL	118.1 ± 66.2	7	101.6 ± 57.0	5	144.4 ± 93.9	5	134.0 ± 97.9	3
HDL cholesterol level, mg/dL	52.1 ± 15.5	17	48.9 ± 14.2	18	39.2 ± 12.9	10	42.0 ± 19.1	10
SBP, mm Hg	134.9 ± 17.4	16	130.5 ± 20.6	13	134.7 ± 23.5	8	127.1 ± 23.2	4
DBP, mm Hg	80.2 ± 8.7		79.8 ± 10.2		84.3 ± 12.9		81.2 ± 15.1	
Fasting glucose level, mg/dL	94.5 ± 17.3	2	95.2 ± 23.0	1	104.4 ± 37.8	2	107.6 ± 62.5	1

Abbreviations: SD, standard deviation; HDL, high-density lipoprotein; SBP, systolic blood pressure; DBP, diastolic blood pressure.

a Metabolic syndrome is defined as any 3 of the following: waist circumference >102 cm (men) or >88 cm (women), triglyceride level ≥150 mg/dL, HDL cholesterol level <40 mg/dL (men) or <50 mg/dL (women), blood pressure ≥130/85 mm Hg, or fasting glucose level ≥100 mg/dL.

b The change in values was significant (*P* < .05) for waist circumference (women and men) and SBP (men only).

**Table 3:** Effect of an 8-Week Diet and Exercise Intervention on Indexes of Insulin Resistance, Hillsdale United Methodist Church Wellness Study, Advance, North Carolina, April-August 2008

Parameter	Women (n = 27), Mean ± SD			Men (n = 14), Mean ± SD		

	Baseline	Week 8	*P* Value^a^	Baseline	Week 8	*P* Value^a^
Insulin level, µU/mL	7.0 ± 3.9	4.3 ± 4.4	<.001	20.1 ± 22.8	14.6 ± 18.8	.04
HOMA^b^	1.6 ± 1.0	1.1 ± 1.0	<.001	5.1 ± 5.2	3.7 ± 4.6	.04
HbA1c, %	6.0 ± 0.7	5.5 ± 0.6	<.001	5.9 ± 0.3	5.7 ± 1.8	.006
SCD1^c^	0.09 ± 0.03	0.07 ± 0.02	<.001	0.08 ± 0.03	0.06 ± 0.02	.01
Palmitoleic acid level, mg/dL	5.9 ± 3.5	4.4 ± 2.5	.01	5.2 ± 2.9	4.4 ± 2.6	.11

Abbreviations: SD, standard deviation; HOMA, homeostatic model assessment; HbA1c, hemoglobin A1c; SCD1, stearoyl-coenzyme A desaturase 1.

a Pairwise analysis of baseline vs week 8 differences for each group (women, men) by using paired *t* test (2-tailed).

b Calculated as (mmol/L fasting glucose × μU/mL fasting insulin)/22.5.

c SCD1 activity was calculated as ratio of serum levels of C16:1 to C16:0 fatty acids
